# Giant panda twin rearing without assistance requires more interactions and less rest of the mother—A case study at Vienna Zoo

**DOI:** 10.1371/journal.pone.0207433

**Published:** 2018-11-28

**Authors:** Martina Heiderer, Carmen Westenberg, Desheng Li, Hemin Zhang, Doris Preininger, Eveline Dungl

**Affiliations:** 1 Tiergarten Schönbrunn GmbH, Vienna, Austria; 2 Wolong Nature Reserve Administration Bureau, Dujangyan City, PR China; Sichuan University, CHINA

## Abstract

The giant pandas’ (*Ailuropoda melanoleuca*) reproductive strategy is unique among mammals. Yet there are characteristics of giant panda behaviour we do not fully understand. Probably one of the least understood is the assumption that in captivity virtually all giant panda females rear only one cub when twins are born and abandon the other if given the chance. So far, only two females have raised twins simultaneously, but just with intensive human assistance. This case-study marks the first successful rearing of giant panda twins in captivity entirely by the mother. Using video data for detailed behavioural observations, we provide the first behavioural assessment of a giant panda female raising two cubs simultaneously without direct human assistance or disturbance. We compared the maternal behaviour during the denning period of twin cubs raised in 2016 with two singleton cubs born 2007 and 2010. YANG YANG, the dam, rested less and interacted more with the twins than with the singletons in the first month postpartum and invested a greater part of her daily time budget on rearing the twins. We discuss potential favourable factors for the autonomous twin-rearing of a female giant panda, which could serve as a model for similar efforts elsewhere.

## Introduction

The reproduction strategy of giant pandas (*Ailuropoda melanoleuca*) is unique among mammals and has been a key issue of intensive research for decades. Females are seasonal, monoestrual breeders, experiencing only a single three-day period of sexual receptivity per year [1–9] and have the lowest mean litter size of all ursids [10, 11], resulting in an overall low reproduction rate. Giant panda neonates are the most altricial of all eutherian mammals having the smallest neonate-maternal weight ratio (1/900). For several weeks they are blind and furless without the ability to eliminate waste on their own [9, 12].

Finding the right balance between activity and inactivity to meet basic demands of nutrition and energy-saving is crucial for every living organism [13, 14]. As the diet of giant pandas consists exclusively of bamboo, only a high level of food intake (up to 45% of body weight) combined with fast rates of digestive passage (5-11hours) allows giant pandas to meet their nutritional needs, which requires approximately 14 hours of daily foraging [15, 16]. The altricial condition of neonates and a low-nutrition diet create the challenging task of balancing activity and rest for a breeding giant panda female [2, 17, 18]. Raising a cub requires an extremely active role of the dam for lactation, thermoregulation, grooming and bowel and bladder stimulation [2, 12, 15]. Females constantly hold their cubs tightly to their bodies and reduce their activity during the first weeks after parturition [2]. Additionally, they do not leave the den, in which the litter is usually born, until several days after birth resulting in a period of fasting [15, 19]. It is regarded as nearly impossible for females to satisfy the needs of more than one cub and several observations of captive bred twins support this assumption. Whereas some observations report that females care for only one cub and ignore the other twin right from parturition [20], other studies show that females initially try to care for both cubs, but after cradling and grooming their twins for a few hours ultimately give up and rear only one cub [2, 15, 21]. Interestingly nearly all females readily accept either twin if presented to them individually, which led to the current zoo practice of swapping twins back and forth between the mother and a nursery every few days or even hours, so each cub is half mother- and half hand reared [20, 22].

Only two females were reported to pick up and care for both cubs in the case of a twin birth [23–25]. The female QUING QUING from Chengdu Zoo, China, was the first giant panda to raise two cubs simultaneously. The second female MEI MEI from Wakayama Adventure World, Japan, raised two sets of twins successfully. However, in all reported cases the females had human assistance. Although the cubs were not swapped, the keepers hand-fed the female and sat next to the den and constantly observed the female to be able to assist if necessary. If the mother had difficulties picking up both cubs, or repositioned herself and could potentially roll over a cub, the keepers picked up the cub and repeatedly assisted the female. Nevertheless, the predominant number of abandoned twin cubs in captivity, led to the widely accepted assumption that panda females are not capable of raising more than one cub simultaneously [2, 11, 15, 20]. However, some rare and anecdotal reports of wild female giant pandas [15] give rise to the possibility that females are able to meet the demands of two infants. Furthermore, it would be highly questionable why twinning occurs in about half of all litters, when inevitably only one cub will survive. The birth of a second panda cub may be an insurance in the case one dies [15], a non-adaptive trait inherited from their ursid ancestry, or an outlier from the past, suggesting that ancient pandas living in the centre of their historic range, were more capable to raise twin cubs. In contrast to giant pandas, dams of other ursid species attempt to rear the entire litter, regardless of its size [2, 11].

The requirements crucial for successful rearing of neonates, such as lactation, cleaning and bladder stimulation, cannot be reduced or discontinued without placing the infants´ survival in jeopardy. This supports the hypothesis that twins demand a higher level of activity of females and maternal investment increases compared to raising a singleton. In all bear species the altricial cubs are usually born in a secluded den and females undergo a fasting period after parturition to meet the cub’s needs [11, 26]. Most bear species can increase their food intake from 8.000kcal/day to as many as 20.000kcal/day, which allows them to store enough fat for hibernation or fasting during the denning period. With a year-round average energy expenditure of about 3.500kcal/day and a food intake of approximately 5000kcal/day a panda can in contrast obtain only enough digestible energy to store a small amount of fat [11, 27, 28]. Thus, reduced activity observed in giant panda females in the time after parturition may be important to compensate the low energy intake. Resting times have to be kept constant during rearing and might even demand to be increased with twins. Accordingly, females would need to reduce interactions with infants or other behaviours to be able to increase rest during twin rearing and behavioural differences should be obvious compared to singletons.

In this study we provide the first evidence that a female giant panda is capable of raising two cubs simultaneously without human assistance. The birth and the acceptance of both cubs by the dam YANG YANG at Vienna Zoo, Austria provided the unique opportunity to analyse maternal care behaviour and mother-infant interactions. The study focused on the denning period, crucial for altricial infant survival [29, 30] lasting approximately three to four months after parturition. To understand the requirements of female investment and maternal care during twin and singleton rearing we compared mother-infant interactions and analysed general time budgets of denning behaviours of two single- and one twin birth in the Vienna Zoo. Differences or similarities in rearing of twins compared to singletons should help to identify important factors for twin-rearing and explain why giant pandas hardly ever accept both infants in a twin birth.

## Material and methods

### Ethics statement

The behavioural observations were performed without physical contact with the study animals. The observation protocol in this article complies with the current laws of Austria, the country in which the study was performed. The study adhered to the Animal Behaviour Society guidelines for the use of animals in research, ethical approval was not required and data collection methods were approved by the responsible curator and director of the Vienna Zoo. Animal husbandry, care and welfare of the study species in the Vienna Zoo strictly adhere to the EAZA (European Association of Zoos and Aquaria) Standards for the Accommodation and Care of Animals in Zoos and Aquaria.

### Subjects and housing

**Fig 1:**
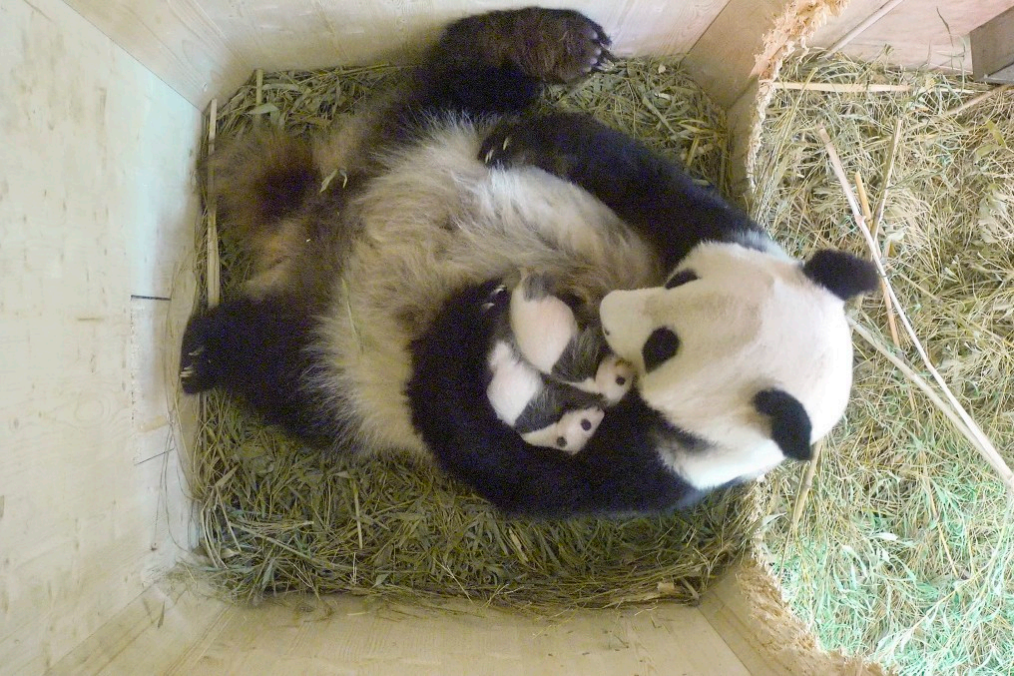
Female giant panda (*Ailuropoda melanoleuca*) with 3 week old twins in the denning box in the Vienna Zoo.

The behavioural study focused on a female (YANG YANG, studbook #514) and a set of twins (male FU BAN, studbook #1026 and female FU FENG, studbook #1027) born at Vienna Zoo in 2016 (Fig 1). The female YANG YANG was born in 2000 in Wolong and was transferred to Vienna in 2003 together with a male giant panda (LONG HUI, studbook #526) of the same age. Until 2016 YANG YANG raised overall 5 cubs (male FU LONG, studbook #685 in 2007, male FU HU, studbook #789 in 2010, male FU BAO, studbook #887 in 2013 and the twins FU FENG and FU BAN in 2016) which were sired by the male LONG HUI in natural mating.

All giant pandas are housed in an exhibit at Vienna Zoo, comprising a 135m^2^ indoor enclosure, an approx. 1000m^2^ outdoor enclosure and 33.5m^2^ off-exhibit rooms. All cubs were born in an artificial denning box, a wooden box located in one of the off-exhibit rooms. The first and the second singleton cub stayed in the denning box throughout the entire observation period of 100 days. The twin cubs were transferred to a hollow tree located in the indoor enclosure by the female 71 and 72 days after parturition. The adult male, and sire of the cubs, was separated from the female and had no contact to the cubs throughout the entire observation period.

### Data collection and analysis

All observations were made using stored video data from an infrared sensitive digital video observation system (Bosch, Germany). Two cameras, a microphone and infrared lights were used to record behaviours 24 hours a day, over a total of 42 days. One camera and microphone were located in the ceiling of the denning box (Fig 1). The other camera was located in the artificial hollow tree. Video data analysis was limited to observations of activities in the denning box and the hollow tree trunk.

The study focused on nine general behavioural states, three mother-only and 10 mother-infant behaviours in regard to maternal care and mother-infant interaction. An ethogram (Table 1) was developed using existing behavioural ethograms for giant pandas (sensu [31, 32, 33]) and was modified according to results of previous observations at Vienna Zoo [34]. The video data were analysed using the focal animal sampling method with an instantaneous scan interval of one minute (IS). Scan samples were used to estimate the percentage of time the focal panda performed various behaviours and to record social distance between all individuals. All-occurrence focal sampling (AO) was used to record the frequencies of different social behavioural events (repositioning of female, repositioning of the cub, licking, squirming and lactation) and vocalizations of the twins [35, 36]. For social distance measurements between the female and each cub IS were assigned to four categories: ‘full contact’ between the mother and the cubs; ‘ground contact’, when the cubs lay on the ground in contact with the female; ‘intermediate vicinity’ was assigned when the cubs lay on the ground next to the female without contact and the category ‘three adult body length’ was chosen when the female was outside the breeding den.

**Table 1:** Ethogram of behavioural categories for general behaviours, mother-only and mother-infant behaviours of a female giant panda at Vienna Zoo. IS represents instantaneous scan interval sampling and AO all-occurrence focal sampling.

Super Category	Category	Code	Definitions	Recording
General behaviour	Rest	IA	Lying, sitting or standing without changing position.	IS
	Motion	M	Changing from one position to another, e.g. change between sitting and lying.	IS
	Locomotion	L	Any kind of directional travel between points including walking, running and climbing.	IS
	Ingestion	Ing	Water intake and feeding of any kind of food (bamboo, vegetables and fruit) including both consumption and handling of food.	IS
	Interaction	Int	All behaviours listed below under Mother-Infant.	IS
	Partially Exposed	PE	Infant is partially covered by the mother's hand, arm or head and it is impossible to determine which behaviour should be scored because of poor view.	IS
	Hidden	H	Infant is completely covered by the mother's hand, arm or head	IS
	Not Visible	NV	Animal moves temporarily out of view.	IS
	Other	O	Other behaviours than mentioned above.	IS
Mother-Infant	Nursing	S	Visible suckling movements of the cubs´ mouth.	AO
	Positions-Paw	POP	Transfers infant from hand to the other or moves infant to another position on body using her paw. Also includes when female pushes infant with hand toward nipples into position for suckling.	AO
	Positions-Mouth	POM	Transfers infant from hand to the other or moves infant to another position on body using her mouth. Also includes when female pushes infant with muzzle toward nipples into position for suckling.	AO
	Comforts	CO	Mother provides physical comfort to infant as a response to its vocalization or movement	AO
	Delayed Comforts	DCO	Mother provides physical comfort at least 5 seconds and no more than 1 minute after the infant vocalizes or moves.	AO
	Slight movement	SM	Mother moves single body parts without changing position	AO
	Change position	CP	Changing from one position to another, e.g. change between sitting and lying	AO
	Picks Up-Paw	PUP	Mother picks up infant from the ground with paws.	AO
	Picks Up-Mouth	PUM	Mother picks up infant from the ground with mouth.	AO
	Drops infant	DROP	Mother drops infant on the ground. Infant must “freefall” or slide very rapidly.	AO
	Groom	L	Mother licks the infant´s genital region, abdomen, back, mouth etc.	AO
	Other Interactions	OI	Any other form of interaction between the mother and the infant	AO
Mother-Only	No Comforts	NCO	Mother shows no response to infant vocalization or movement	
	Departs	DPS	Leaves infant by moving at least one adult panda body length away or by leaving the den while the infant remains behind.	AO
	Approaches	AP	Returns to den or approaches to within 1 body length.	AO

Data collection started on day six after parturition, at the point when both cubs could be observed, and continued to day 28, including the complete third (day 15–21) and fourth (day 22–28) week after parturition. To investigate behavioural changes over time, the eighth (day 50–56) and 12^th^ (day 78–84) week were analysed. Video data of twin rearing comprised 888 h of video footage which were compared with behavioural data from the same female collected in the same manner in the third, fourth, eighth and 12^th^ week postpartum of single cubs, reared in 2007 and 2010. As the dam usually fed outside the den, except from few occasions when she brought bamboo branches into the den after a feeding bout, feeding was not statistically analysed.

To describe similarities and differences of rearing periods and behavioural changes over time, mean percentages and standard errors (± SE) of behavioural categories per day were calculated. We compared percentages of respective behaviours (interaction, resting, nursing, out of den) displayed by the female in week three, four, eight or 12 during the cub rearing periods in 2007, 2010 and 2016 using Generalized Linear Mixed Models (GLMMs). The GLMMs allow repeated measurements per day and week to be fitted in the model as random variables. The statistical assumptions for GLMM analysis were met (Kolmogorov-Smirnov test). The percentages of respective behaviours of one particular week (3, 4, 8 or 12) were entered as dependent variables, with the rearing period (2007, 2010 and 2016) as predictor variable. We entered the identities of rearing period (days of respective week) as nested random variables. For post-hoc tests we used Student’s t Statistic with sequential Bonferroni correction for alpha because of repeated pairwise comparisons of rearing periods. GLMMs were additionally used to calculate differences in exposure time between twins and singletons. Percentages of either not-, partially or fully exposed during week 3, 4, 8 or 12 were entered as dependent variable with cubs (2007, 2010, FF-2016 and FB-2016) as predictor variable. We entered the identities of cubs (days of respective week) as nested random variables. We also used GLMMs to compare repositioning events performed by the female to move the twin cubs. The percentages of paw and mouth repositioning per day for week two, three, four, eight or 12 were entered as dependent variable, with repositioning mode (paw and mouth) as predictor variable. We entered the identities of repositioning mode (days of respective week) as nested random variable. Statistical analysis was performed using SPSS version 23 (SPSS Inc., Chicago, IL, USA).

## Results

The twin cubs were born on August 7^th^ 2016, in the off-exhibit denning box. The mother protected the cubs from the environment with her arms or by sitting in a curled position. At first only one cub was discovered. The twins were observed simultaneously for the first time on day 6 postpartum. From the 12^th^ day, both cubs were regularly visible.

### Maternal behaviour

The mother first left the den on the eighth day postpartum, but early bouts of absence were brief. Her first extended bout outside the den occurred on the 19^th^ day postpartum lasting 23 minutes. Whereas the time spent outside the den was very limited in the first month after parturition (<7% of her time budget), the female spent an increasingly greater portion of her day out of the den in the eighth (34.68±4.63%) and the 12^th^ week (62.83±1.57%).

YANG YANG exhibited a period of reduced activity postpartum as she rested (sitting and lying) on average 81.74±2.78% of the instantaneous scans on day six and seven, and at least 69.05±1.10% of the time each day until the fourth week. The female rested in the den 50.50±3.94% per day in the eighth and 28.14±1.74% in the 12^th^ week. The female had two typical resting postures, a curled sitting posture with her head resting on the ground in front of her or on her hind legs and a lying posture where one whole side of her body rested on the ground. When lying on the side she often embraced her hind legs with the forepaws.

On average the female spent 23.65%±0.68% of time per day to interact with the cubs in the third and fourth week. Interactions decreased to 12.92%±0.89% in week eight and 7.47%±0.53% in week 12. The interactions mainly consisted of grooming the cubs and repositioning herself or the cubs. Twenty-five percent of grooming occurred during or within 10 minutes after suckling bouts and nearly 50% of the cubs’ vocalizations or restless movements were responded with grooming by the female.

**Fig 2:**
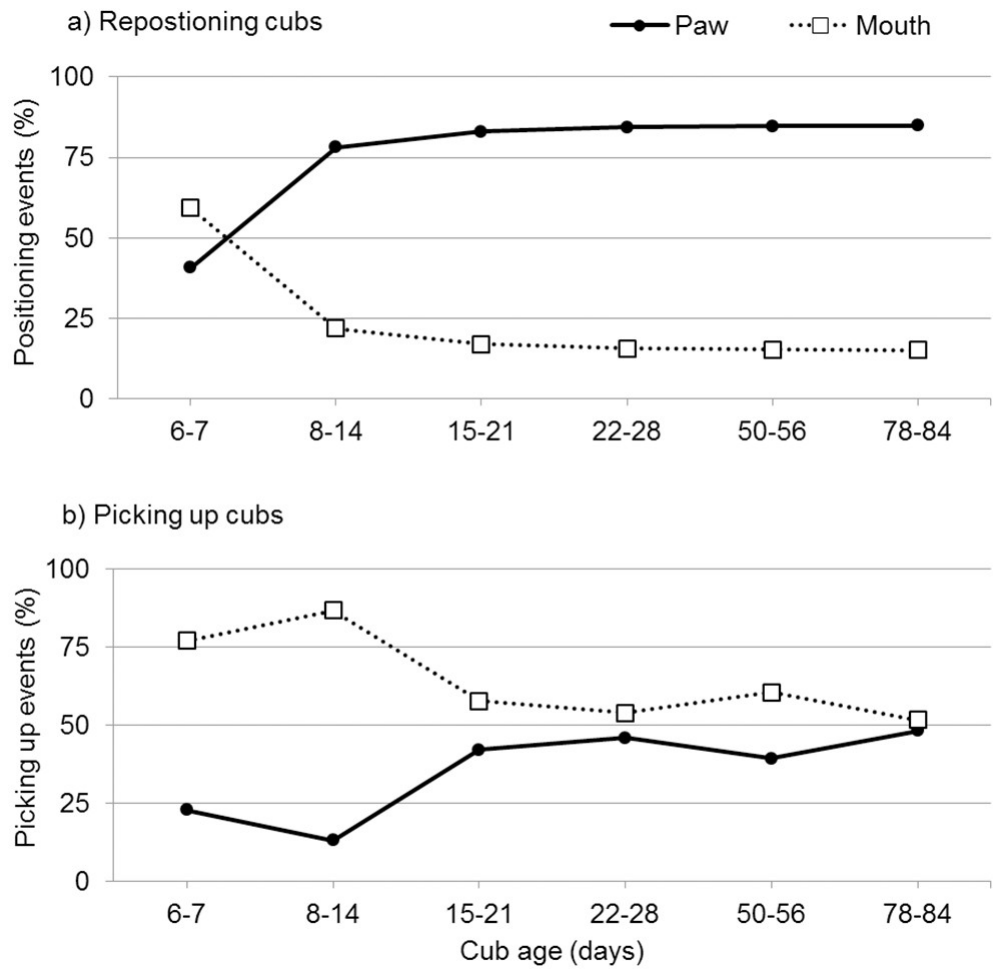
Maternal behaviour of giant panda female YANG YANG repositioning (a) or picking up (b) twin cubs at Vienna Zoo.

When the female repositioned the cub to facilitate resting or lactation, but also as a response to the cubs´ vocalizations, she used the mouth more often than the paws in the first two days of observation. Differences in repositioning method were also found in the following weeks (GLMM: F_1,12_ = 147.168; P < 0.001), but already from the second week on she used the paws distinctively more often (GLMM: pairwise comparison: *ß* = 56.366; SE = 4.646; *t* = 12.131, *P* < 0.001 Fig 2a). When the female returned to the den during the first 18 days postpartum she mainly picked up the cubs from the ground using her mouth and when using her paw the female often used her muzzle to stabilize the cub in the paw. As soon as the female started to leave the den more regularly and had to pick up the cubs more often, she used both her mouth and her paws equally (Fig 2b). Usually one cub was picked up with her mouth whereas the other one was lifted from the ground by the female’s paws. Overall we couldn’t find any significant differences in the females´ behaviour towards each individual twin. Licking the cubs, repositioning the cubs or picking them up varied daily, but no tendency to an individual cub could be detected. Moreover the twins spent the same time suckling and no difference in frequency and duration between the cubs was observed (S2 Data).

### Physical contact

**Fig 3:**
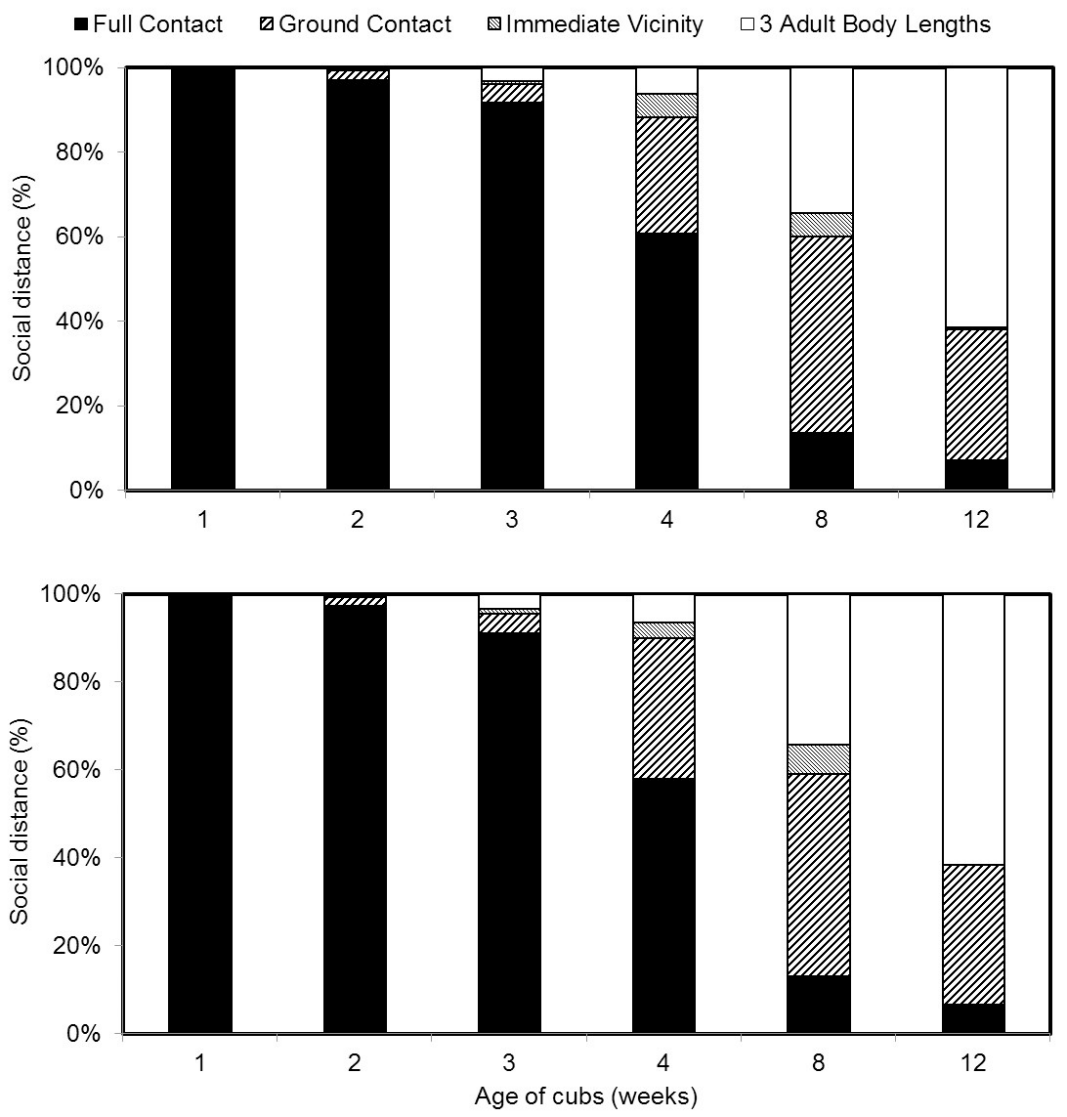
Social distance between mother and twin giant pandas a) FU FENG and b) FU BAN in the first 12 weeks of cubs life. Social distance measured as percentage of 1-minute scan intervals per 24 h.

Contact between the cubs and the dam changed dramatically throughout the first three months, but changes did not differ between the cubs (Mann-Whitney U test: for all social distance categories p>0.05, S3 Data). In the first two weeks the dam usually lay on her side or sat upright in a curled position, holding the cubs with the arms to the belly or chest the so called hand-hold position. During this period, hand-hold contact was observed in nearly 100% of the instantaneous scans. The female picked up the cubs from the ground as soon as possible in week three and four, but allowed the cubs to be in contact with the ground for longer periods of time in week eight and 12. In the eighth week the female mostly laid on her side with the cubs resting beside her, hence the cubs were more frequently in ground contact than in full contact or intermediate vicinity (Kruskal-Wallis multiple pairwise comparisons tests: p<0.05 for both cubs). As the twins aged and the dam spent less time in the den, the most frequent recorded contact level was ‘three adult body lengths’ between the twins and the female (Fig 3a and 3b).

### Comparison of single- and twin rearing

The mother did not leave the den until the eighth day postpartum with her first singleton, as soon as the fourth day with her second and on the eighth day with the twins. Her time spent outside the den was similarly limited in all rearing periods throughout the third week after parturition (Table 2). Interestingly, there was a distinct difference in the onset of feeding behaviour of YANG YANG between the rearing periods. Whereas short bouts of feeding were already observed from the 10^th^ day postpartum in 2007 and the eighth day in 2010, it was not until the 17^th^ day after the twin birth the female started feeding in 2016.

**Table 2:** Comparisons of behavioural time budgets of the giant panda female YANG YANG behaviours are reported in average percent of time per day during week 3, 4, 8 and 12 after parturition.

	Rear. Per.	Time (%) ± SE					
		Day	Day	Day	Day	Day	Day
		6–7**	8–14**	15–21	22–28	50–56	78–84
Rest	2007	98.33±0.35	93.01±0.26	86,49±1.35^a^^,^^b^	77.62±0.98^b^	29.18±4.06^a^^,^^b^	26.76±4.46^a^
	2010	/	/	81.10±0.67^a^^,^^c^	76.41±0.51^c^	55.26±3.11^a^	38.90±1.81^a^^,^^c^
	2016	81.74±2.78	75.82±0.93	70.77±1.02^b^^,^^c^	69.05±1.10^b^^,^^c^	50.50±3.94^b^	28.14±1.74^c^
Interact	2007	0.42±0.07	2.38±0.5	5.11±0.67^a^^,^^b^	10.20±0.74^a^^,^^b^	6.18±0.60^a^^,^^b^	2.49±0.50^b^
	2010	/	/	14.06±0.76^a^^,^^c^	17.30±0.51^a^^,^^c^	9.21±0.61^a^^,^^c^	3.20±0.22^c^
	2016	15.41±2.22	20.59±1.25	25.18±0.44^b^^,^^c^	22.12±1.02^b^^,^^c^	12.91±0.89^b^^,^^c^	7.47±0.53^b^^,^^c^
Out of den	2007	0.17±0.18	1.23±0.36	1.96±0.61	3.41±0.44^a^^,^^b^	59.37±4.63^a^^,^^b^	66.19±4.56
	2010	/	/	1.86±0.52	2.03±0.41^a^^,^^c^	31.31±2.76^a^	55.11±1.90
	2016	0.00	0.30±0.06	2.12±0.70	6.57±0.45^b^^,^^c^	34.68±4.63^b^	62.83±1.57
Lactate	2007	/	/	3.83±1.36^b^	6.57±1.17^b^	5.00±0.27^a^	1.99±0.32^b^
	2010	/	/	4.04±0.67^c^	4.14±0.45^c^	2.64±0.25^a^^,^^c^	1.87±0.20^c^
	2016	/	/	6.09±0.77^b^^,^^c^	7.44±0.57^b^^,^^c^	4.91±0.44^c^	3.07±0.23^b^^,^^c^

GLMM: p<0.05 for pairwise comparison

^a^2007 vs. 2010,

^b^2007 vs. 2016,

^c^2010 vs. 2016

** not statistically analysed

**Fig 4:**
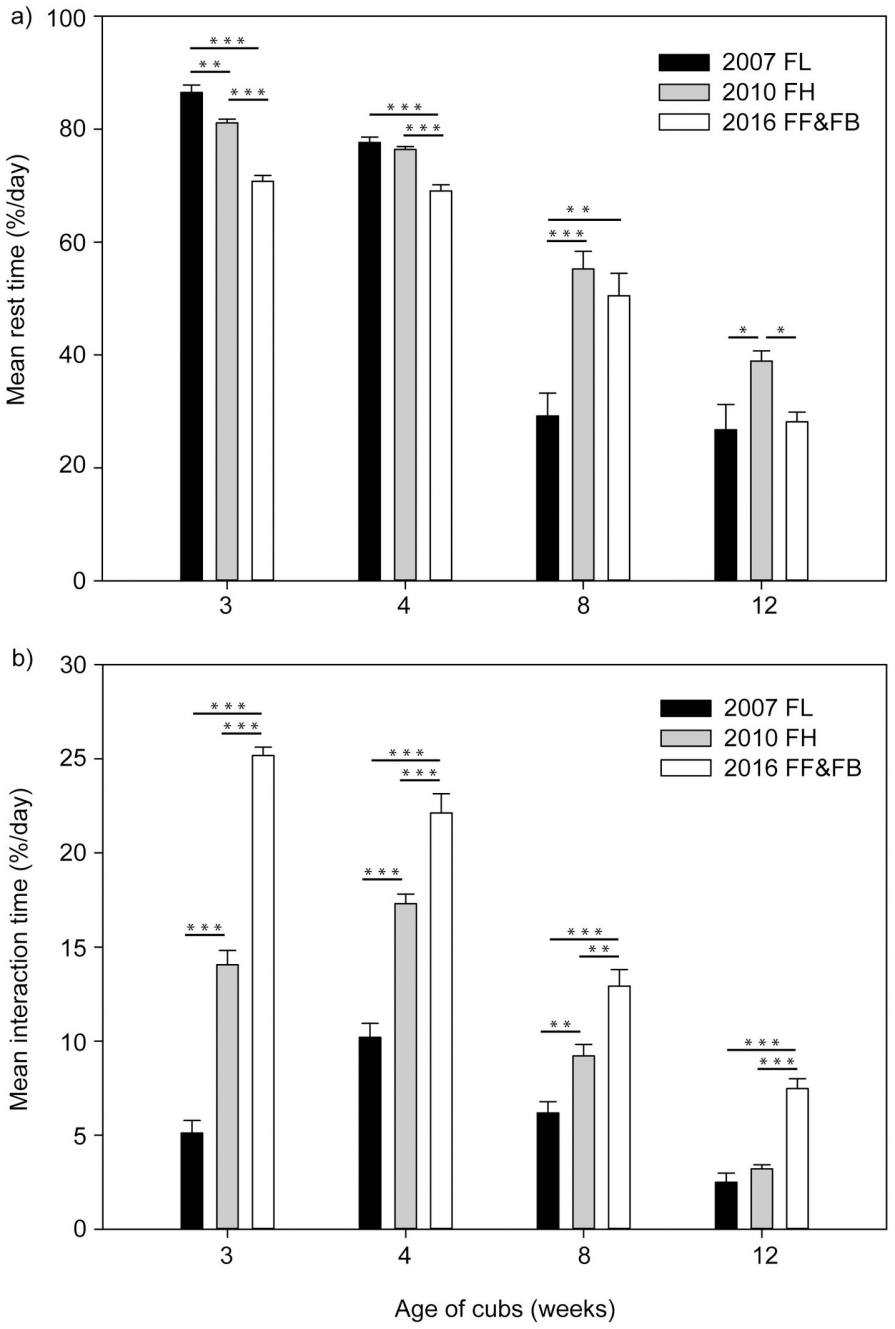
Comparisons of a) resting behaviours, b) mean interactions of the giant panda female YANG YANG with the single cubs FU LONG (FL) 2007, FU HU (FH) 2010 and the twins FU FENG (FF) & FU BAN (FB) 2016) during the first 12 weeks after parturition.

On average the female rested less during the third and the fourth week after the twin birth compared to the same period of both singleton rearing in 2007 (GLMM week 3: pairwise comparison: *ß* = -15,713; SE = 1.481; *t* = -10.608, *P* < 0.001; GLMM week 4: pairwise comparison: *ß* = -8.569; SE = 1.275; *t* = -6.720, *P* < 0.001; Table 2) and 2010 (GLMM week 3: pairwise comparison: *ß* = -10.327; SE = 1.481; *t* = -6.972, *P* < 0.001; GLMM week 4: pairwise comparison: *ß* = -7.359; SE = 1.275; *t* = -5.771, *P* < 0.001; Fig 4a). In week eight of twin rearing resting times were higher than rest periods in 2007 and similar to rest periods in 2010, whereas in week 12 no differences were observed between resting times in 2007 vs. 2016, but the mother rested more during this period in 2010 compared to 2016 (Table 2).

YANG YANG spent more time interacting with the twins than with the single cubs raised in 2007 and 2010 (Fig 4b) in all analysed weeks and mother and twin interactions declined continuously over the first 12 weeks (Table 2).

**Fig 5:**
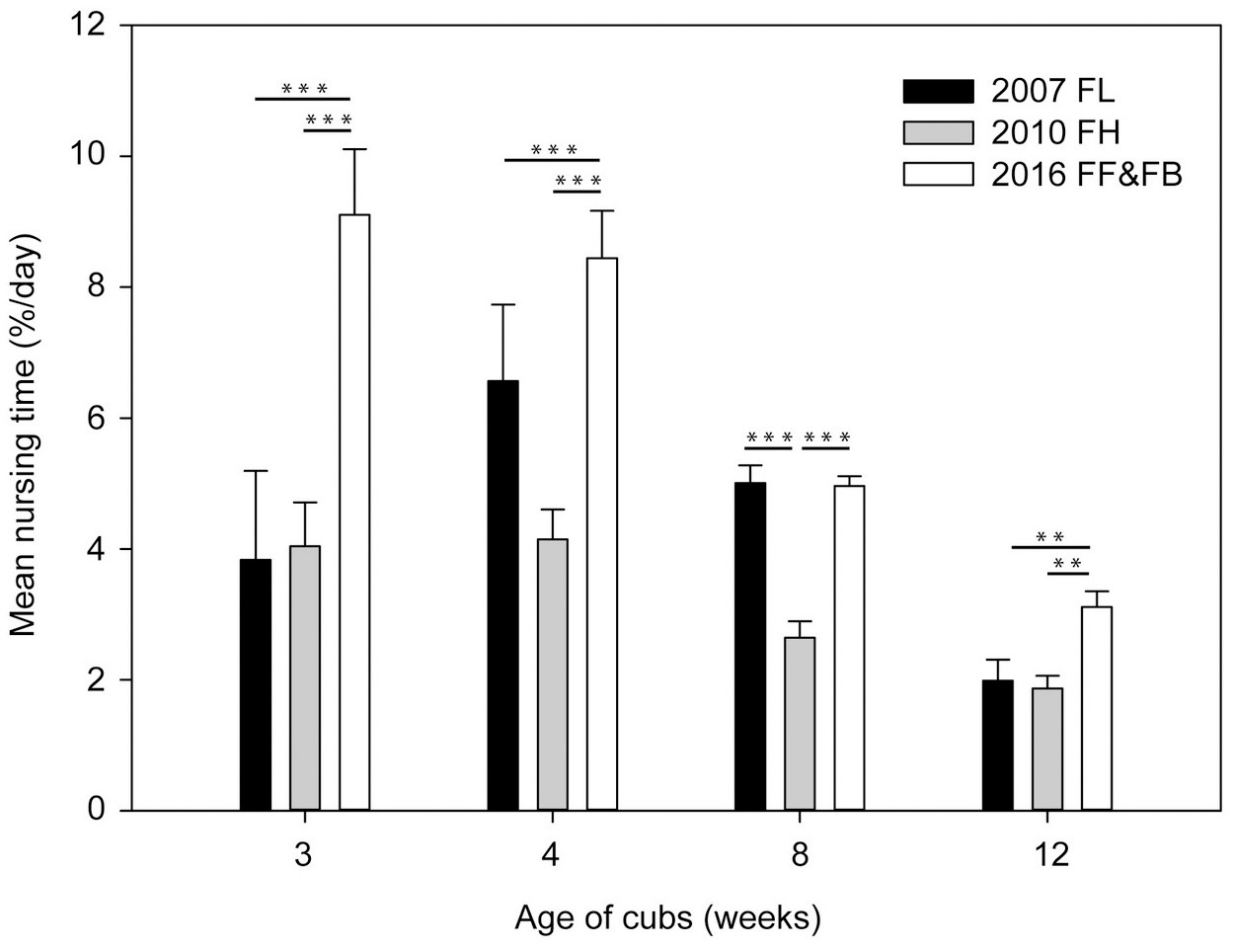
Comparisons of lactation of the giant panda female YANG YANG of the single cubs FU LONG (FL) 2007, FU HU (FH) 2010 and the twins FU FENG (FF) & FU BAN (FB) 2016) during the first 12 weeks after parturition.

In her active time the female spent twice as much time suckling the twins compared to singletons in the first month after parturition (GLMM week 3: pairwise comparison 2016 vs. 2007: *ß* = 4.780; SE = 0.830; *t* = 5.762, *P* < 0.001; pairwise comparison 2016 vs. 2010: *ß* = 3.849; SE = 0.830; *t* = 4.639, *P* < 0.001; week 4: pairwise comparison 2016 vs. 2007: *ß* = 3.561; SE = 0.727; *t* = 4.897, *P* < 0.001; pairwise comparison 2016 vs. 2010: *ß* = 3.771; SE = 0.727; *t* = 5.186, *P* < 0.001; Fig 5). Nursing times of twins remained higher compared to singleton nursing in 2010 during the entire observation period. In week eight the mother spent similar times nursing the cub raised in 2007 compared to twins, while in week 12 the twins were nursed more (Fig 5).

Already in the first days after parturition the dam protected the twins from the environment by covering them with her arms, paws or body just as observed in previous years with singletons. However, the twin cubs were more often fully exposed in week 3 (<40%), week 4 (<70%) and week 8 (<90%) than the singletons (Table 3). By week 12 singletons and twins showed similar exposure (GLMM: F_3,24_ = 1.830; P = 0.169) and were no longer shielded by the dam.

**Table 3:** Comparisons of exposure of cubs FU LONG 2007, FU HU 2010 and FU FENG (FF) & FU BAN (FB) 2016. Behaviours are reported in average percent of time per day during week 3, 4, 8 and 12 after parturition.

	Rearing Period		Time (%) ± SE					
			Day	Day	Day	Day	Day	Day
			6–7**	8–14**	15–21	22–28	50–56	78–84
Not exposed	2007		83.70±2.55	82.46±1.65	71.05±2.78^a^^,^^b^	37.94±3.44^a^^,^^b^	3.29±0.72^b^	2.32±1.16
	2010		/	/	46.62±6.91^a^	11.17±1.46^a^	2.54±0.39	1.06±0.30
	2016	FF	89.25±2.19*	63.82±5.82	30.34±3.72^b^	12.25±2.24^b^	0.95±0.14^b^	1.38±0.24
		FB		65.09±5.28	33.63±4.32^b^	9.97±1.69^b^	1.07±0.18^b^	1.36±0.29
Partially exposed	2007		6.88±0.45	6.82±1.23	15.25±2.06^a^^,^^b^	24.11±1.32^a^^,^^b^	20.79±2.45^b^	5.86±2.99
	2010		/	/	33.91±4.90^a^	51.93±3.62^a^^,^^c^	21.31±1.03c	8.44±0.69
	2016	FF	8.04±.1.33*	16.95±2.63	26.83±1.50^b^	15.84±1.21^b^^,^^c^	5.16±0.89^b^^,^^c^	2.41±0.22
		FB		17.46±3.05	25.47±1.02	14.68±1.84^b^^,^^c^	5.12±0.28^b^^,^^c^	4.51±0.60
Fully exposed	2007		9.42±2.10	10.71±1.11	13.69±1.80^b^	37.95±2.59^b^	75.81±3.01^b^	91.81±3.49
	2010		/	/	19.47±2.45^c^	36.89±2.74^c^	76.14±1.29^c^	90.50±0.91
	2016	FF	2.59±0.89*	19.77±3.27	42.77±4.40^b^^,^^c^	71.90±2.48^b^^,^^c^	93.89±0.93^b^^,^^c^	96.16±0.30
		FB		18.02±2.29	40.95±14.81^b^^,^^c^	75.32±3.11^b^^,^^c^	93.81±0.39^b^^,^^c^	94.04±0.75

GLMM: p<0.05 for pairwise comparison

^a^2007 vs. 2010,

^b^2007 vs. 2016,

^c^2010 vs. 2016

* FF and FB combined

** not statistically analyzed

## Discussion

The giant panda female YANG YANG at Vienna Zoo successfully raised twins without any human assistance. Particularly during the first month after parturition of the twins we observed distinct differences in the behaviour of the mother compared to singleton rearing. The female rested less, interacted more and spent more time lactating the twin cubs. Lactating twins required nearly twice as much time as nursing singletons, which of course sounds reasonable. However, lactation is considered the most energetically demanding component of maternal care [37, 38] and the female should allocate her resources conservatively to ensure her own survival. The constraints imposed by an exclusive bamboo diet are typically quoted as central factor in the reproductive strategy of giant pandas. Large mammals like bears usually use stored nutrients for milk production, which allow them to give birth and lactate at sites and times independent from food resources [39]. Considering the additional time per day necessary to feed two juveniles, less resting time remained for the female, as at this stage even short discontinuities in care of the cubs could jeopardize them [2]. Maternal investment also included mother-infant interactions with the twins comprising of grooming, picking them up and repositioning the cubs which also required more time than during singleton rearing. Only giant pandas, and to some extend sun bears (*Helarctos malayanus*) and spectacled bears (*Ursus ornatus*) show a high level of maternal care [18, 19, 26, 40]. Most northern bear species demonstrate a rather passive maternal behaviour [11, 41]. A study on Japanese black bears (*Ursus thibetanus japonicus*) found no clear difference in the overall maternal investment for a single cub and twin cubs [42]. Litter size and cub survival of other ursids are affected by differing factors. Polar bear cub survival is mainly influenced by maternal mass and cub mass, but litter size is unimportant [43]. In brown bears (*Ursus arctos*) females may abandon single cub litters to increase overall fitness in the following breeding season [44]. It thereby remains difficult to draw across species comparisons. Nevertheless, our observations oppose the assumption that the altricial condition of the neonate makes it impossible for the female to provide both neonates with the same level of care [2, 15]. The current case-study demonstrates, if only exceptionally, that it is possible for a giant panda female to raise twins entirely on her own and provide similar care for each twin.

Subsequently we propose several factors which might have made this remarkable behaviour of the female possible. First, we suggest that a careful protection of the female against all stressors during the early post-partum period is crucial. Providing an environment enabling a close mother-infant contact may be extremely important in insuring constant maternal care. Our results demonstrate that during the first month the female is almost constantly in full contact or at least body contact while the cubs are on her side on the ground and spends at least eight days continuously in the denning box. This behaviour emphasized the importance of denning areas, a shielded environment out of public view. Moreover a natural breeding environment equipped with an off-exhibit breeding room, or off-exhibit denning-box can provide a protected habitat. Abandonment of cubs or whole litters in other bear species is often related to human disturbance [45–47]. Even though captive giant pandas are used to noise and presence of humans, comprising daily husbandry routine and direct interactions during training, enclosure design and regular interference likely have negative effects on maternal behaviour. Thereby, the Vienna Zoo practices a hands-off approach in managing the giant pandas. In all rearing periods YANG YANG chose the most secluded breeding option, the wooden denning box in the off-exhibit area.

Secondly we suggest that previous and repeated experience of single births may have prepared YANG YANG for the twin birth and may have increased the probability of appropriate maternal behaviour. Generally multiparous females are more suited to provide even two cubs simultaneously with the right amount of maternal care rather than primiparous females [10, 23, 30]. Hence, we believe that experienced mothers could be more successful to rear twins if given the opportunity.

Overall also the physiological condition respectively the trade-off between energy intensive direct maternal care and the dams’ energy reserves has to be considered. Due to the specialized diet, giant panda females are not able to undergo significant increases in weight before denning and do not fast as long as most other bears [11]. Despite a likely better physical condition of captive giant pandas, due to constant food supply and veterinary care, until now females have never reared twins entirely by themselves in captivity. After the twin birth YANG YANG however fasted nine to 10 days longer than during singleton rearing while providing comfort and warmth to the cubs, which again demonstrates the costs and necessary investment of rearing twins. Generally reproductive performance can be increased with supplementary food in a variety of species (e.g. [48, 49]). In European hares (*Lepus europaeus*) leveret growth and survival are enhanced when females are fed an energy rich diet [50] and females raise a larger litter size than hares fed on a low-fat diet (pers. Com. W. Arnold). During all reported giant panda pregnancies food was not enriched until after parturition and postpartum fasting period when the female was offered nutritional enhanced bread (including e.g. chopped bamboo leaves, bamboo stem flour, rice flour, corn flour, oat flakes, vegetables, quail eggs, oil and salt) in addition to bamboo. Therefore, rearing of panda twins cannot be explained by a supplemented diet.

Lastly another contributing factor may be the physical development of the cubs at birth. Cubs from twin litters often seem to show a great disparity in body weight and development (see [2]. Studies throughout several taxa have shown that hatching-size respectively birth weight is associated with early mortality as neonates of lower body weight have a lower survival rate (e.g. [51, 52–54]). Hence, giant panda maternal investment and–care of twin litters could be dependent on differences or rather similarities of juvenile size and weight at birth. Due to the hands-off management at the Vienna zoo the twins were not measured at birth but appeared very similar in body weight and size proportions as soon as they could have been observed via camera (personal observation). First measurements were performed 80 days after parturition demonstrating only minor differences in body weight of the twins (4.26 and 3.97 kg) and correspond to other juvenile measurements at this age [12, 20]. Contrary to sex differences reported from Bejing Zoo [12] the female cub in the Vienna Zoo was and remained slightly heavier than its male twin.

The majority of observations, research studies and zoo-rearing demonstrate an absolute litter size of one cub and further studies should enhance the current investigations on factors benefiting twin rearing. We thereby call upon conservation institutions to attempt to let giant panda dams raise their offspring without assistance and closely document habitat conditions and maternal behaviour.

## Conclusion

This study marks the first successful rearing of giant panda twins entirely by the mother, and highlights the increased maternal investment necessary during the first month after parturition. We propose multiple contributing factors for this successful twin rearing such as the physiological preconditions of both cubs, the experience of YANG YANG as a multiparous mother and the secluded and highly undisturbed breeding environment. Although this study demonstrates that it is possible to rear twins without human assistance, the current sample size requires follow up studies to see if our findings can be supported and to confirm or identify crucial factors for a successful twin acceptance and -rearing.

## Supporting information

S1 Data Time budgets. Daily time budgets of all analysed behaviours, and animals.
(XLSX)

S2 Data Suckling duration and frequency. Daily duration and frequency of lactation of the twin giant panda cubs born 2016 in the Vienna Zoo.
(XLSX)

S3 Data Social distance. Daily percentages of social contact categories between the mother and the twin giant panda cubs born 2016 in the Vienna Zoo.
(XLSX)

S4 Data Exposure. Daily percentages of exposure categories of all four analyzed cubs are presented.
(XLSX)

S5 Data Maternal behaviour. Daily percentage of repositioning or picking up twin cubs by the female.
(XLSX)
